# Supplementary material for: Ideometrics: a scientific approach to generating, evaluating, and prioritising ideas
Source: J Glob Health. 2025 Dec 19;15:04360. doi: 10.7189/jogh.15.04360 (PMC12904029; doi:10.7189/jogh.15.04360)
Supplement: Online Supplementary Document [file jogh-15-04360-s001.pdf]

**Supplement to: Rudan I, Sheikh A. Ideometrics: a scientific approach to generating, evaluating, and prioritising ideas. J Glob Health. 2025;15:04360.**

**Box S1.** Addressing epistemic risks explicitly, such as over-generalisation, value-ladenness, or the limits of quantification, when unifying qualitative and quantitative traditions to preempt possible critiques of reductionism.
**Box S2.** A discussion of ethical, equity, and governance implications, such as algorithmic bias in AI-based ideation, ownership of collective knowledge, and participatory fairness.
**Box S3.** A schematic conceptual model illustrating the relationships and feedback loops among idea generation, evaluation, and prioritisation.
**Box S4.** Position on including non-Western epistemologies.
**Box S5.** The potential biases in LLM-generated ideas (e.g., hallucination or cultural skew) and their relevance to „value of information".
**Box S6.** Explaining LLM's research priority setting within the CHNRI exercise.
**Box S7.** The role of political authority, financial power, deliberate political propaganda and disinformation in ideometrics.
**Box S8.** Recognising and appropriately managing reliance on lead author's prior work.
**Table S1.** Scientific field(s) of origin and application for ideometrics methods.
**Table S2.** Comparative attributes of methods within ideometrics field of science.

**Box S1.** Addressing epistemic risks explicitly, such as over-generalisation, value-ladenness, or the limits of quantification, when unifying qualitative and quantitative traditions to preempt possible critiques of reductionism.

Any attempt to unify methodological traditions that span qualitative, quantitative, participatory, and expert-driven approaches must be sensitive to epistemic risks such as over-generalisation, value-ladenness, or the limits of quantification. This paper is intentionally designed not to reduce these traditions to a single metric or normative framework, but rather to recognise their differences while placing them into a shared conceptual architecture.

Our aim is not to homogenise, simplify, or quantify all traditions under a single evaluative logic. Instead, ideometrics seeks to map diversity without erasing it, by showing that: (i) diverse methods share a structural sequence (generation, evaluation, prioritisation), but differ profoundly in epistemology, values, and appropriate contexts; (ii) provide a comparative framework, not a reductionist one, thus enabling readers to understand when a qualitative approach is better suited than a quantitative one, or vice versa; (iii) explicitly acknowledge value-ladenness, as many evaluation tools encode normative assumptions (e.g., equity, feasibility, evidentiary standards) and ideometrics aims to expose these assumptions, not obscure them; (iv) avoid over-generalisation, by systematically classifying methods according to structure, complexity, prior-information requirements, and participation modes (e.g., **Tables S1 and S2s**), which demonstrates their heterogeneity; (v) respect the limits of quantification, because quantitative methods are powerful, but not universally applicable; ideometrics highlights entire families of purely qualitative and deliberative techniques (e.g., citizen juries, Open Space, JLA, dialectical methods) to ensure that pluralism is preserved.

In summary, ideometrics does not suggest that all methods should be unified substantively or mathematically; it merely unifies them conceptually, by recognising that all traditions, qualitative or quantitative, are ultimately attempting to address the same three fundamental questions about ideas: (i) How to generate them? (ii) How to evaluate them? (iii) How to prioritise them?

**Box S2.** A discussion of ethical, equity, and governance implications, such as algorithmic bias in AI-based ideation, ownership of collective knowledge, and participatory fairness.

The integration of social, computational, and participatory approaches raises significant ethical, equity, and governance considerations, particularly regarding algorithmic bias, ownership of collective knowledge, and fairness in deliberative processes. These concerns are increasingly central to contemporary research ethics and will undoubtedly play a critical role in the future development of ideometrics.

We have intentionally not expanded these topics in this foundational manuscript for two key reasons: (i) Maintaining conceptual clarity and scope discipline; this paper's primary aim is to articulate the conceptual foundations, historical roots, and integrative architecture of ideometrics. Introducing a comprehensive ethics framework here would substantially broaden the manuscript and risk overshadowing the core argument that establishes ideometrics as a scientific field; (ii) A dedicated follow-up work is already underway, focusing specifically on the ethical, equity, and governance dimensions of ideometrics.

This work will include: algorithmic bias and fairness in AI-supported ideation and prioritisation; ownership, credit, and intellectual responsibility in expert-crowdsourced and community-generated idea pools; participatory equity, power asymmetries, and democratic legitimacy; transparency, reproducibility, and accountability in idea-evaluation processes; frameworks for ethical oversight bodies and research institutions; and other related questions. These topics require a level of detail that is beyond the already extensive scope of the current manuscript.

Importantly, ideometrics is explicitly designed to be compatible with diverse epistemological and ethical commitments. Its purpose is not to prescribe uniform value structures, but to provide a conceptual architecture within which different ethical frameworks, e.g., participatory, consequentialist, deontological, justice-oriented, or algorithmic, can be transparently articulated and compared. Thus, the omission of detailed ethical analysis in the current manuscript reflects scope limitation, not conceptual oversight.

**Box S3.** A schematic conceptual model illustrating the relationships and feedback loops among idea generation, evaluation, and prioritisation.

A schematic conceptual model illustrating the relationships and feedback loops among idea generation, evaluation, and prioritisation will be valuable when developed. It will be essential for the operationalisation of ideometrics, as the field evolves. However, we intentionally did not include such a figure in this foundational manuscript for three reasons:

(i) Premature formalisation would risk oversimplification: at this early stage, any schematic model that attempts to depict data flows, interdependencies, or feedback loops would risk implying a fixed architecture or normative structure for ideometrics. One of the key arguments of the paper is that the field is incomplete, pluralistic, and evolving. A prematurely formal diagram could unintentionally suggest determinism or closure.

(ii) The present paper aims to be conceptual, not operational: this article establishes ideometrics as a scientific field by mapping the historical, methodological, and epistemological terrain. Its core contribution is integrative and conceptual. A fully operational model, showing feedback systems, iteration cycles, and methodological pathways, belongs to the next stage of the development of ideometrics, when empirical and computational work can support more definitive formalisation.

(iii) A dedicated follow-up work will provide the operational framework, focusing specifically on the operationalisation of ideometrics, including data flows between generation, evaluation, and prioritisation; iterative loops (e.g., evaluation, reformulation, regeneration); decision architectures for human–machine co-evaluation; process maps for institutions applying ideometrics; and formal conceptual models suitable for teaching, governance, or software systems. Including these detailed models here would significantly expand the manuscript and dilute its purpose as a conceptual foundation.

**Box S4.** Position on including non-Western epistemiologies.

Idea-generation, evaluation, and consensus-building traditions exist in every culture, and non-Western epistemologies, such as African palaver systems, Indigenous consensus practices, and East Asian deliberative philosophies, offer rich and underutilised resources for understanding how ideas are generated and validated. However, we would like to clarify that the present manuscript deliberately focuses on methodological traditions that have already formalised themselves in the scientific literature as named, documented, and structured methods.

Our key criterion for inclusion of methods in this, initial paper, was the existence and the possibility for identification, through Google or Google Scholar search, of a key reference that could be associated as the theoretical origin of each method. Our aim in this foundational paper is to synthesise these referenced and operationalised methods into a single conceptual framework for ideometrics. Because many non-Western traditions have historically been transmitted orally, embedded in social practice, or articulated through broader philosophical systems rather than discrete methodological protocols, they require a different form of treatment. Such treatment would be unlikely to fit structurally within the current manuscript, without substantial expansion and theoretical adaptation.

To avoid superficiality and to treat these traditions with the seriousness and rigor that they deserve, we believe it is preferable to dedicate a separate, follow-up work specifically to: (i) systematically mapping non-Western and Indigenous idea-generation and consensus frameworks; (ii) analysing their underlying epistemic commitments; (iii) examining how they intersect with or challenge Western-derived methods; (iv) and exploring how ideometrics can incorporate them in a respectful, rigorous, and epistemically pluralistic manner. Including only several examples here, without full contextual explanation, risks misrepresenting these traditions by compressing them into Western methodological categories. We are, therefore, cautious not to reduce rich cultural epistemologies to brief illustrations in a paper already operating under strict conceptual scope.

**Box S5.** The potential biases in LLM-generated ideas (e.g., hallucination or cultural skew) and their relevance to „value of information".

| |
|---|
| AI-based ideation tools introduce important considerations, particularly regarding bias, cultural skew, and the interpretability of machine-generated suggestions, that merit critical examination. However, the subsection on "Approaches to Ideation in the Digital Age" was not meant to provide an exhaustive or technical review of AI architectures, nor to discuss their limitations in depth. Rather, it was designed to serve a mapping function, demonstrating that computational approaches represent another major methodological lineage feeding into ideometrics.<br><br>The aim was to show where AI fits in the broader conceptual framework, not to provide a comprehensive critique of machine-learning methodology. A deeper analysis of LLM hallucinations, training-data biases, alignment challenges, fairness constraints, and the empirical literature on AI-supported priority-setting, while extremely valuable, would substantially extend the manuscript and shift the balance away from its core purpose: establishing the conceptual foundations of ideometrics and synthesising the historically diverse traditions of idea generation, evaluation, and prioritisation.<br><br>We acknowledge that LLMs and other AI systems bring risks of hallucination, cultural skew, and training-data biases, and that these factors influence the reliability and "value of information" of AI-generated ideas. Therefore, integrating AI with human evaluative and deliberative processes is important, and we already pioneered in this area by comparing the outputs of the CHNRI method to that of LLMs.<br><br>Also, a dedicated follow-up work will examine the role of AI in ideometrics in greater depth, including empirical evidence from global health priority-setting, human–machine hybrid evaluation models, algorithmic fairness frameworks, and ethical governance implications. |

**Box S6.** Explaining the first LLM's research priority setting within the CHNRI exercise [76].

Within the reference [76], when prompted, on 28 July 2023, to: "please define and list, in order of importance, the greatest research priorities for global pandemic preparedness", ChatGPT generated the list using:

(1) Latent semantic space reasoning, as its primary mechanism: it reasoned across its internal representation of scientific literature, pandemic experiences, global health structures, and conceptual relationships. This reasoning occured in a high-dimensional latent space, where patterns from millions of texts have been compressed into conceptual vectors. This allowed ChatGPT to infer: (i) what types of research are normally prioritised in pandemics; (ii) what global health frameworks emphasise; (iii) what challenges LMICs commonly face; (iv) and how major institutions (WHO, CDC, CEPI, GAVI, etc.) are described in expert discourse. Thus, the broad categories (vaccines, surveillance, supply chains, One Health, social sciences) emerged from statistical associations across global health knowledge. This is why its output appeared "theoretical" and "general", as the authors noted, because latent representations generalise across contexts rather than anchoring themselves in LMIC-specific implementation issues.

(2) Simulation of human expert knowledge, as its secondary mechanism: ChatGPT also approximated how global health experts typically talk and think, because its training includes: (i) WHO reports; (ii) academic consensus documents; (iii) CHNRI-related papers that the authots authored previously; (iv) pandemic preparedness literature; (v) One Health and global governance discourse. This did not simulate any particular expert, but it did simulate the collective tone and structure of expert knowledge. That is why ChatGPT placed vaccine development first (a very standard consensus position), surveillance second or third (another universal priority), governance and supply chains in the top five, behavioural sciences in the mid-range, and ethical/economic analyses towards the end. This reflected the "shape" of expert reasoning, without formal scoring.

3. Importantly, ChatGPT did not perform any comparative evaluation against predefined criteria. Therefore, unlike the CHNRI scorers, it did not: (i) use explicit criteria such as feasibility, equity, or potential for paradigm shift; (ii) apply a scoring system; (iii) attempt to mimic the 5-criterion weighting; or (iv) compute RPS or AEA-e scores. ChatGPT's ordering of priorities emerged implicitly from: (i) semantic strength in training data; (ii) frequency with which certain themes are presented as top priorities; (iii) conceptual centrality in its knowledge graph. Thus, its ranking reflected statistical salience, not criteria-based evaluation.

The outputs aligned very well with CHNRI because CHNRI is designed to capture collective human knowledge, and a large-scale language model is also a compression of collective human knowledge; the divergence also revealed mechanisms, as humans emphasised LMIC operational challenges, while ChatGPT emphasised universal theoretical concerns, mirroring "experience" vs. "knowledge".

**Box S7.** The role of political authority, financial power, deliberate political propaganda and disinformation in ideometrics.

Clearly, the factors such as political authority, financial power, deliberate political propaganda and disinformation fundamentally determine which ideas are prioritized and implemented in the real world (e.g., funding decisions, censorship). A field positioning itself as a science of prioritization must grapple with these core, non-technical determinants. These dynamics are critical sub-domains within the ideometrics research agenda, rather than external factors. Also, ideometrics' aspiration to „assist societies" must confront how power asymmetries (e.g., funding biases in global health) skew prioritisation. Prioritisation processes are inevitably shaped by structural incentives and power. Ideometrics needs to explicitly recognise these influences as part of its research agenda, and that the forthcoming work will operationalise these issues through case studies and empirical analysis.

The influences mentioned above all profoundly influence which ideas are prioritised, adopted, or suppressed in real-world settings, thus shaping the "selection environment" within which ideas compete. Therefore, they are highly relevant to the long-term development of ideometrics. Our intention in the present manuscript was not to dismiss these dynamics, but to signal that they lie beyond the analytic scope of this initial, theoretical, concept-defining paper, and they will become more relevant in the follow-up work that will focus on implementation.

The purpose of this work is to establish the foundational architecture of ideometrics by mapping historical methods and articulating the core cognitive stages of idea generation, evaluation, and prioritisation. A full treatment of political economy and disinformation would require extensive conceptual tools from political science, sociology, media studies, and critical theory, including Foucault's work, that could not be incorporated without substantially expanding and reorienting the manuscript.

Indeed, one of the ambitions of ideometrics is to enable systematic study of how non-technical determinants shape idea selection. Rather than treating these issues as "external," we will consider them as core subdomains within the future ideometrics research agenda. In the follow-up work, we will include the analysis of political incentives, power asymmetries, media and platform dynamics, disinformation ecosystems, funding structures, and institutional decision logics. A dedicated follow-up work will focus specifically on these socio-political determinants, using case studies from global health, research funding, and public decision-making, integrating historical frameworks such as COHRED, CHNRI, and deliberative democratic models, thus positioning power, politics, and disinformation as central research questions for ideometrics, not peripheral exclusions.

**Box S8.** Recognising and appropriately managing reliance on lead author's prior work.

The manuscript cites several examples from the lead author's prior work, especially regarding CHNRI and related priority-setting methodologies. However, this should not be interpreted as insularity, but rather as an unavoidable and appropriate feature of a foundational paper in a field where significant conceptual and methodological advances have, to date, emerged from a relatively small set of pioneering contributions. The reasons why these references are necessary and scientifically justified are:

(i) Historical accuracy: The development of structured, transparent, and reproducible idea-evaluation frameworks in global health is historically tied to the CHNRI tradition. Any rigorous account of how ideation, evaluation, and prioritisation methodologies evolved must accurately represent this lineage, regardless of authorship;

(ii) Representative examples: The manuscript does not rely on these works as exclusive evidence; rather, they are presented as illustrative cases within a much broader ecosystem of methods from engineering, psychology, management, philosophy, economics, innovation studies, and AI. Supplementary Tables alone show more than 70 methods and more than a dozen scientific fields represented, i.e., far beyond the lead author's contributions.

(iii) Foundational relevance to ideometrics: The CHNRI method, and related work on structured expert scoring, transparency, and reproducibility, has played a unique role in demonstrating the feasibility of comparing idea-evaluation methods systematically, and throughout the entire cycle of generating, evaluating and prioritising ideas. It thus provides a concrete empirical foundation for proposing ideometrics as a new scientific field.

Excluding it would weaken, not strengthen, the conceptual argument. The manuscript draws on the lead author's prior work only where historically or conceptually necessary; this work is presented alongside, and explicitly integrated with, a broad array of independent methodological traditions; and ideometrics is clearly not derived from a single method or lineage, but from the synthesis of more than seventy methods across over a dozen scientific fields. We therefore believe that the current level of reference to prior work is both proportionate and essential for historical and conceptual completeness, while the manuscript as a whole demonstrates clear engagement with a wide and diverse scholarly landscape.

**Table S1.** Scientific field(s) of origin and application for ideometrics methods*.

| Method / Approach | Primary Scientific Field(s) of Origin | Typical Domains of Application |
|---|---|---|
| Stream of consciousness / Freewriting | Psychology; Literary theory; Education | Creative writing; Idea generation |
| TRIZ | Engineering; Patent science; Systems theory | Product design; Industrial engineering |
| Morphological analysis | Astrophysics; Systems engineering | Scenario planning; Technology design |
| Lateral thinking | Psychology; Cognitive science | Creativity; Strategy |
| SCAMPER | Education; Creativity studies | Business innovation; Ideation workshops |
| Mind mapping | Cognitive psychology; Education | Learning; Planning; Brainstorming |
| Heuristics & biases framework | Cognitive psychology; Behavioural economics | Decision science; Public policy; Risk |
| Six Thinking Hats | Psychology; Organisational science | Group thinking; Ideation; Facilitation |
| Brainstorming | Advertising; Organisational psychology | Innovation; Marketing; R&D |
| Focus groups / In-depth interviews | Sociology; Anthropology; Qualitative research | Market research; Public health; Policy |
| Delphi method | Military foresight; Decision science | Forecasting; Research priorities |
| Nominal Group Technique | Organisational psychology; Public health | Consensus-building; Priority-setting |
| World Café | Organisational development; Participatory design | Strategy; Stakeholder dialogue |
| Open Space Technology | Organisational culture; Anthropology | Change management; Crisis response |
| InnoCentive & open innovation | Pharmaceutical R&D; Innovation economics | R&D challenges; Open science |
| James Lind Alliance (JLA) | Health services research; Public engagement | Patient-led research prioritisation |
| CHNRI method – idea generation | Global health; Epidemiology; Public policy | Health R&D priority-setting |
| IdeaScale / crowdsourcing | Innovation management; Digital participation | Government; Industry; Civic tech |
| Human-Centred Design | Engineering; Psychology; Design research | Product & service design |
| Design thinking | Design science; Management; Engineering | Innovation; Startups; Education |
| Agile ideation sprints | Software engineering; Organisational design | Product development; Strategy |
| Hackathons | Computer science; Open-source culture | Tech innovation; Healthcare; Policy |

| Method / Approach | Primary Scientific Field(s) of Origin | Typical Domains of Application |
|---|---|---|
| Lean startup methodology | Management science; Entrepreneurship | Innovation strategy; Product-market fit |
| Genetic algorithms | Computer science; Evolutionary biology | Optimisation; Robotics; AI |
| Automated hypothesis generation | Medical informatics; AI; Library science | Biomedical discovery; Knowledge discovery |
| Generative adversarial networks | Machine learning; Computer vision | Creative AI; Simulation; Art |
| LLMs for ideation support | AI; Computational linguistics | Science; Strategy; Design |
| Peer review | Philosophy of science; Academic publishing | Research evaluation |
| Modified Delphi scoring | Decision science; Policy analysis | Health & tech prioritisation |
| Consensus conferences | Health policy; Evidence synthesis | Guidelines; National recommendations |
| AHP (Analytic Hierarchy Process) | Mathematics; Operations research | Complex decisions; Investment priorities |
| Cost-effectiveness analysis | Health economics; Welfare economics | Health policy; Resource allocation |
| Cost-benefit analysis | Economics; Public policy | Infrastructure; Regulation; Planning |
| Net Present Value (NPV) | Finance; Economics | Capital investment |
| Internal Rate of Return (IRR) | Finance; Business economics | Capital allocation; Project evaluation |
| Patent metrics & analysis | Innovation economics; Information science | Tech forecasting; Industrial strategy |
| Bibliometrics (citations, JIF, h-index) | Scientometrics; Information science | Research evaluation; Academic policy |
| Technology Readiness Levels (TRLs) | Aerospace engineering; Innovation management | Technology development; Funding |
| Multi-criteria decision analysis (MCDA) | Decision science; Operations research | Health tech assessment; Policy |
| SWOT analysis | Strategic management; Business studies | Corporate planning; Non-profit strategy |
| Weighted scoring models | Operations research; Management science | Technology selection; Procurement |
| Pugh Matrix | Mechanical engineering; Product design | Engineering concept selection |
| FDV (feasibility–desirability–viability) | Design thinking; Innovation science | Product strategy; Social innovation |
| Wisdom-of-the-crowd | Statistics; Behavioural science | Forecasting; Rating; Open ranking |

| **Method / Approach** | **Primary Scientific Field(s) of Origin** | **Typical Domains of Application** |
|---|---|---|
| Prediction markets | Economics; Game theory | Policy modelling; Innovation prediction |
| JLA evaluation stages | Public engagement; Health research | Research agenda-setting |
| CHNRI evaluation stage | Global health; Epidemiology | Priority-setting for R&D |
| Altmetrics | Information science; Scientometrics | Research communication |
| Logical consistency & deductive reasoning | Philosophy; Logic | Theoretical evaluation |
| Empirical testability / falsifiability | Philosophy of science; Experimental science | Hypothesis evaluation |
| Paradigm shift analysis (Kuhn) | History & philosophy of science | Field evolution; Epistemology |
| Paired comparison methods | Psychology; Operations research | Ranking alternatives |
| Multi-voting / dot voting | Participatory design; Facilitation | Workshops; Policy co-creation |
| RAND/UCLA Appropriateness Method | Clinical epidemiology; Biostatistics | Medical appropriateness decisions |
| Essential National Health Research (ENHR) | Health systems; Development economics | National priority-setting |
| GRADE & Evidence-to-Decision frameworks | Evidence-based medicine; Epidemiology | Guidelines; Clinical research |
| Combined Approach Matrix (CAM) | Development economics; Public policy | Priority-setting for LMICs |
| Real Options Analysis | Finance; Mathematical modelling | Innovation under uncertainty |
| R&D portfolio matrices (BCG, GE) | Strategic management; Industrial economics | Corporate R&D; Product strategy |
| Stage-Gate model | Innovation management; Engineering | Technology development cycles |
| Knowledge graphs | AI; Semantic web; Computational linguistics | Scientific discovery; Data integration |
| Reinforcement-learning optimisation | AI; Control theory | Strategy optimisation; R&D pipelines |
| Automated priority setting via LLMs | AI; Decision science | Research & policy prioritisation |
| Citizen juries | Political science; Deliberative democracy | Public policy; Ethics |
| Public consultations | Governance; Sociology | Policy development |
| Participatory budgeting | Political science; Urban governance | Municipal finance; Policy |

| Method / Approach | Primary Scientific Field(s) of Origin | Typical Domains of Application |
|---|---|---|
| Occam's Razor | Philosophy; Scientific method | Theory choice |
| Bayesian inference | Statistics; Probability theory | Decision-making; Evidence evaluation |
| Dialectical method | Philosophy; Critical theory | Theory development |
| Epistemic humility & pluralism | Epistemology; Ethics | Science governance; Policy |

*This table was developed in response to comments from several reviewers, aiming to clarify the unifying nature of ideometrics by exposing primary fields of origin and typical domains of application of the identified ideometrics methods. However, in some cases, there are no clear-cut answers and some cells are therefore based on arbitrary decisions by the lead author, to ensure the completeness of the table.

**Table S2.** Comparative attributes of methods within ideometrics field of science.*

| **Method / Approach** | **Participatory mode** | **Prior information required** | **Degree of structure** | **Facilitates comparison?** | **Multifactorial (uses multiple criteria)** | **Technical complexity** | **Cognitive complexity** |
|---|---|---|---|---|---|---|---|
| Stream of consciousness / Freewriting | Individual | None | Low | No | Low | Very low | Low |
| TRIZ (Theory of Inventive Problem Solving) | Expert / team | Moderate | High | Yes | High | High | High |
| Morphological analysis | Expert / team | Moderate | High | Yes | High | Moderate | High |
| Lateral thinking | Individual / small group | Minimal | Moderate | No | Low | Low | Moderate |
| SCAMPER technique | Individual / group | Minimal | Moderate | No | Low | Low | Low–Moderate |
| Mind mapping | Individual / group | Minimal | Moderate | Partial (via visual clustering) | Low–Moderate | Low | Low–Moderate |
| Heuristics and biases framework | Individual / expert | Minimal | Moderate | Partial (via debiasing comparisons) | Moderate | Low | Moderate–High |
| Six Thinking Hats | Group | Minimal | High | Partial (via separate perspectives) | Moderate | Low | Moderate |
| Brainstorming | Group | None | Low | No | Low | Very low | Low |
| Focus groups & in-depth interviews | Participatory group | Minimal | Moderate | No (primarily exploratory) | Moderate | Moderate | Moderate |
| Delphi technique (general) | Expert panel | Moderate–High | Very high | Yes | High | Moderate | High |

| Method / Approach | Participatory mode | Prior information required | Degree of structure | Facilitates comparison? | Multifactorial (uses multiple criteria) | Technical complexity | Cognitive complexity |
|---|---|---|---|---|---|---|---|
| Nominal Group Technique (NGT – idea generation phase) | Participatory group | Minimal | High | Partial (through initial ranking) | Moderate | Low–Moderate | Moderate |
| World Café | Participatory group | Minimal | Low–Moderate | No (primarily dialogic) | Low | Low | Low–Moderate |
| Open Space Technology | Participatory group | None | Low | No | Low | Very low | Low–Moderate |
| InnoCentive / open-innovation challenges | Broad, distributed participants | Moderate (problem brief) | High | Yes (via judging/scoring) | Moderate | High (platform) | Low–Moderate (for solvers) |
| James Lind Alliance (JLA – idea generation) | Strongly participatory (patients, carers, clinicians) | Minimal | High | Partial (shortlisting) | Moderate | Moderate | Moderate |
| CHNRI method – idea generation (4D framework) | Expert crowdsourcing | Moderate–High | Very high | Yes (across avenues/options/questions) | High | Moderate–High | High |
| IdeaScale & similar platforms | Broad participatory | None | Moderate–High | Yes (via votes/scores) | Low–Moderate | Moderate–High | Low–Moderate |
| Human-Centred Design | Participatory / co-design | Minimal–Moderate | Moderate | Partial (prototypes vs needs) | High (desirability, feasibility, viability, etc.) | Moderate | Moderate |

| Method / Approach | Participatory mode | Prior information required | Degree of structure | Facilitates comparison? | Multifactorial (uses multiple criteria) | Technical complexity | Cognitive complexity |
|---|---|---|---|---|---|---|---|
| Design Thinking (as ideation framework) | Participatory / multidisciplinary | Minimal–Moderate | Moderate | Partial | High | Moderate | Moderate |
| Agile ideation sprints | Team-based | Minimal–Moderate | High | Partial (selection of tested ideas) | Moderate | Moderate | Moderate |
| Hackathons | Teams / communities | Minimal–Moderate | Moderate | Partial (judging criteria) | Moderate | Moderate | Moderate–High (time pressure) |
| Lean startup methodology (build–measure–learn) | Team / entrepreneurial | Moderate | High | Yes (ideas vs metrics) | High (multiple performance criteria) | Moderate | Moderate–High |
| Genetic algorithms & evolutionary computation | Expert / algorithmic | Moderate | High | Yes | High | High | High (for designers; low for end user) |
| Automated hypothesis generation (literature-based discovery) | Algorithmic / expert user | High (large corpora) | High | Yes | High | High | Moderate–High |
| Generative adversarial networks for idea synthesis | Algorithmic | High (training data) | High | Partial (via discriminator) | Low–Moderate | High | Low (for user) |

| **Method / Approach** | **Participatory mode** | **Prior information required** | **Degree of structure** | **Facilitates comparison?** | **Multifactorial (uses multiple criteria)** | **Technical complexity** | **Cognitive complexity** |
|---|---|---|---|---|---|---|---|
| Large language models (LLMs) for ideation support | Human + AI | None–Minimal | Low–Moderate | Partial (if prompted to compare) | Low–Moderate | High | Low (for user) |
| Peer review | Expert | High | Moderate–High | Partial (across manuscripts) | High (novelty, validity, impact, etc.) | Moderate | High |
| Modified Delphi for scoring | Expert panel | Moderate–High | Very high | Yes | High | Moderate | High |
| Expert panels & consensus conferences | Expert / stakeholder panel | High | High | Yes | High | Moderate | High |
| Analytic Hierarchy Process (AHP – evaluation use) | Expert / group | Moderate | Very high | Yes | Very high | High | High |
| Cost-effectiveness analysis (CEA) | Expert | High | Very high | Yes | High | High | High |
| Cost-benefit analysis (CBA) | Expert | High | Very high | Yes | High | High | High |
| Net present value (NPV) | Expert | High | High | Yes | Low–Moderate | High | Moderate |
| Internal rate of return (IRR) | Expert | High | High | Yes | Low–Moderate | High | Moderate |
| Patent metrics (citations, | Expert / algorithmic | High | High | Yes | Low–Moderate | High | Moderate |

| Method / Approach | Participatory mode | Prior information required | Degree of structure | Facilitates comparison? | Multifactorial (uses multiple criteria) | Technical complexity | Cognitive complexity |
|---|---|---|---|---|---|---|---|
| originality, generality) | | | | | | | |
| Bibliometric & scientometric indices (citations, JIF, h-index) | Expert / algorithmic | High | High | Yes | Low–Moderate | High | Moderate |
| Technology Readiness Levels (TRLs) | Expert | High | Very high | Yes (maturity levels) | Moderate | High | Moderate |
| Multi-criteria decision analysis (MCDA – general) | Expert / group | Moderate–High | Very high | Yes | Very high | Moderate–High | High |
| SWOT analysis | Individual / group | Low–Moderate | Moderate | Partial (between options) | Moderate | Low | Moderate |
| Weighted scoring models | Expert / group | Moderate | High | Yes | High | Moderate | Moderate–High |
| Pugh Matrix (Decision-Matrix Method) | Team | Moderate | High | Yes | Moderate | Low–Moderate | Moderate |
| Feasibility–Desirability–Viability (FDV) framework | Team / design group | Minimal–Moderate | Moderate | Yes | High (three main domains expanded) | Low–Moderate | Moderate |
| Wisdom-of-the-crowd techniques | Broad crowd | Minimal | Low–Moderate | Yes (via aggregate estimates) | Low–Moderate | Low–Moderate | Low |

| Method / Approach | Participatory mode | Prior information required | Degree of structure | Facilitates comparison? | Multifactorial (uses multiple criteria) | Technical complexity | Cognitive complexity |
|---|---|---|---|---|---|---|---|
| Prediction markets | Crowd / experts | Moderate | High | Yes | Moderate | High | Moderate |
| JLA (evaluation & shortlisting stage) | Patients, carers, clinicians | Minimal | High | Yes | Moderate | Moderate | Moderate |
| CHNRI method – scoring & evaluation | Expert crowdsourcing | Moderate–High | Very high | Yes | High (typically 5+ criteria) | Moderate | High |
| Social media engagement metrics (altmetrics as traction proxy) | Broad crowd | Minimal | Moderate | Partial (relative attention) | Low | Moderate | Low |
| Logical consistency & deductive reasoning | Individual / expert | Minimal | Moderate | Partial (between arguments) | Low–Moderate | Very low | Moderate–High |
| Empirical testability, replicability & falsifiability | Scientific community | Moderate–High | High (as norm) | Partial (between hypotheses) | Moderate | Low–Moderate | High |
| Paradigm shift lens (Kuhnian assessment) | Community / meta-analysis | High | Moderate | Partial (between paradigms) | High | Low | High |

| Method / Approach | Participatory mode | Prior information required | Degree of structure | Facilitates comparison? | Multifactorial (uses multiple criteria) | Technical complexity | Cognitive complexity |
|---|---|---|---|---|---|---|---|
| Paired comparison methods | Individuals / groups | Minimal–Moderate | High | Yes | Moderate | Moderate | Moderate |
| Multi-voting and dot-voting | Participatory group | Minimal | Moderate | Yes | Low–Moderate | Low | Low |
| Delphi with ranking rounds (prioritisation) | Expert panel | Moderate–High | Very high | Yes | High | Moderate | High |
| NGT with voting | Participatory group | Minimal | High | Yes | Moderate | Low–Moderate | Moderate |
| AHP (prioritisation role) | Expert / group | Moderate | Very high | Yes | Very high | High | High |
| RAND/UCLA Appropriateness Method | Expert clinicians | High | Very high | Yes | High | High | High |
| Essential National Health Research (ENHR) framework | Mixed stakeholders | Moderate | High | Yes | High | Moderate | High |
| GRADE + Evidence-to-Decision frameworks | Expert panels / guideline groups | High | Very high | Yes | Very high | High | High |
| JLA Partnerships (final top-10 workshop) | Strongly participatory | Minimal | High | Yes | Moderate | Moderate | Moderate |

| Method / Approach | Participatory mode | Prior information required | Degree of structure | Facilitates comparison? | Multifactorial (uses multiple criteria) | Technical complexity | Cognitive complexity |
|---|---|---|---|---|---|---|---|
| Combined Approach Matrix (CAM) | Mixed stakeholders | Moderate–High | High | Yes | High | Moderate | High |
| CHNRI priority-setting (final scores & ranks) | Expert crowdsourcing | Moderate–High | Very high | Yes | High | Moderate | High |
| Real Options Analysis | Expert | High | High | Yes | High (value, risk, flexibility) | High | High |
| R&D portfolio matrices (BCG, GE-McKinsey) | Senior decision-makers | Moderate–High | High | Yes | Moderate–High | Moderate | Moderate |
| Product roadmapping & prioritisation grids | Cross-functional teams | Moderate | High | Yes | Moderate–High | Moderate | Moderate |
| Stage-Gate Model | Cross-functional governance | Moderate–High | Very high | Yes (go/kill/hold) | High | Moderate–High | Moderate–High |
| Knowledge graphs & semantic similarity clustering | Algorithmic / expert user | High | High | Yes | High | High | Moderate |
| RL-based portfolio optimisation | Algorithmic / expert | High | Very high | Yes | High | Very high | High (for designers) |
| Automated priority setting via LLMs | Human + AI | Minimal–Moderate | High | Yes | High (many criteria can be encoded) | High | Low–Moderate (for user) |

| Method / Approach | Participatory mode | Prior information required | Degree of structure | Facilitates comparison? | Multifactorial (uses multiple criteria) | Technical complexity | Cognitive complexity |
|---|---|---|---|---|---|---|---|
| Cross-cutting philosophical & meta-theoretical approaches | Expert / scholarly community | High | Moderate | Partial (between frameworks) | High | Low | High |
| Citizen juries & deliberative democracy forums | Lay + expert participants | Minimal–Moderate | High | Yes | High | Moderate | High |
| Public consultations & e-surveys with weighting | Broad public | Minimal | Moderate–High | Yes | Moderate | Moderate | Low–Moderate |
| Participatory budgeting | Citizens + officials | Minimal–Moderate | High | Yes | Moderate–High | Moderate | Moderate |
| Occam's Razor | Individual / community norm | Minimal | Low–Moderate | Yes (between explanations) | Low | Very low | Moderate |
| Bayesian inference (as prioritisation logic) | Expert / algorithmic | Moderate–High | High | Yes | High | High | High |
| Dialectical method | Small groups / theorists | Minimal–Moderate | Moderate | Partial (between theses) | Moderate | Very low | High |
| Epistemic humility & pluralism | Community ethos | Minimal | Low–Moderate | Partial (keeps alternatives in play) | High (multiple value perspectives) | Very low | High |

*This table was developed in response to comments from several reviewers, aiming to clarify comparative attributes of methods within ideometrics field of science. However, in some cases, there are no clear-cut answers and some cells are therefore based on arbitrary decisions by the lead author, to ensure the completeness of the table.
